# Role of female teachers of childhood education in directing children towards effective use of smart devices

**DOI:** 10.1007/s10639-022-11481-y

**Published:** 2022-11-29

**Authors:** Ali Ahmad Al-Barakat, Omayya M. Al-Hassan, Rommel Mahmoud AlAli, Mu’aweya Mohammad Al-Hassan, Ramzia Ali Al sharief

**Affiliations:** 1 University of Sharjah, Sharjah, United Arab Emirates; 2 The Hashemite University, Zarqa, Jordan; 3 King Faisal University, Al-Ahsa, Kingdom of Saudi Arabia; 4 Ministry of Education, Irbid, Jordan; 5 Yarmouk University, Irbid, Jordan

**Keywords:** Smart devices, Childhood education, Teaching and learning, Uses of smart devices, Effective learning

## Abstract

This study endeavors to investigate the role of female teachers of childhood education in directing children towards the effective use of smart devices in developing their learning experiences. The sample of the study consisted of 83 female teachers in the northern region of Jordan, who were selected using the available sampling method. To achieve the aim of the study, a semi-structured interview was prepared and its validity and consistency were verified.

The results shown that childhood education female teachers achieved advanced roles in directing children towards the effective use of smart devices, where effective direction towards the use of smart devices was represented in: directing children to self-organize their learning during using smart devices, directing children to acquire digital social interaction skills and directing them to learn innovation during using smart devices, with the importance of directing them to avoid the harms of using smart devices through preventive guidance.

Moreover, the findings of the study revealed the importance of directing children to participate in various digital activities, as well as directing them to learn through digital applications that are purposeful and suitable to their mental capabilities. Based on the research findings, the study presented a number of relevant recommendations.

## Introduction

In the last years of the 21th century, human life has become increasingly associated with information and communication technology (ICT) in light of the wide spread of mobile computing among all human-society categories, including the use of smartphones, electronic reading devices, tablet devices that operate by touch, … etc. Children are considered one of the society categories that started to deal with touch-operated devices (iPads, Blackberry Playbook, Android tablets, … etc.). These devices are deemed to be the most appropriate ones for children usage, since they are low-cost and can be easily used in schools as a more effective educational tool compared with previously used technologies, such as traditional desktop and/or laptop computers (Oliemat et al., 2018; Rambe, 2012; Schindler et al., 2017).

More specifically, Al-Bagdadi (2014) and Hamdi (2007) argued that children prefer to learn with smart devices, since these devices provide knowledge through different electronic websites and with various means and facilitate the learning process in any place and at any time without needing to go out or move from a place to another. Furthermore, it is possible to provide children with direct feedback from teachers and correct their answers more accurately and with better assessment, sending the assessment results in a direct manner.

Furthermore, using touch-based smart devices is consistent with the mental nature of children, since these devices contribute remarkably to enabling children to build knowledge according to their mental, knowledge and social characteristics. Smart devices are considered among the dynamic and interactive means that reinforce the children’s linguistic, mathematical and scientific skills, among others (Papadakis et al., 2021; Sung et al., 2016).

Yadav & Chakraborty (2021) ascertained that children interact with smart devices according to their mental capabilities in an effective and constructive way. Moreover, the children’s linguistic and mental capabilities grow and develop through touching the screens of smart devices. In this context, Nikolayev et al. (2022) found that touch-screen app. supported with language contributes to reinforcing the Theory of Mind (ToM) interactions, through children’s games with embedded voice-overs. This confirms that directing children to use game applications with voice contributes to developing cognitive structures in children, meaning that language grows and develops with practicing digital games by children constructively and interactively, accompanied by discussions with adults.

Based on this, directing children to effective use of smart devices should rely on holding constructive and critical discussions after the use of children of digital applications. This will not be achieved unless in light of using applications that suit children, which means that employing smart devices in the learning of children should be based on their growth characteristics and psychological nature, in terms of choosing applications that suit their ages, which in turn contributes to enabling children to interact with the touchscreens of smart devices with enthusiasm, motivation, pleasure and activity (Gözüm & Kandir, 2021; Grifth & Arnold, 2019; Papadakis et al., 2022).

Also, directing children to use applications of smart devices that suit their nature contributes considerably to developing the child’s character to be socially interactive, and think in an innovative and imaginary manner. For instance, directing children to use video-chatting apps contributes to developing social interactive skills with others, while directing them to use drawing apps and storytelling helps children in practicing innovative, analytical and imaginative thinking and mastering learning new linguistic terms and words, in addition to understanding scientific and mathematical concepts (Wiley et al., 2016; Yadav & Chakraborty, 2021). Furthermore, cause-effect apps reinforce class participation and increase learning independence (Wiley et al., 2016).

Based on the above, previous studies, such as Bergman (2021), Chen (2015) and Hamdi (2008), revealed that smart devices, once correctly used, may achieve various learning outcomes, increase the social interaction opportunities among children and reinforce the chances of children’s participation in learning environments, thereby enabling them to work actively and enthusiastically and helping them understand and assimilate knowledge soundly in their cognitive structures.

In order to achieve high effectiveness in using smart devices in developing the children’s learning experiences, previous literature in education (Azevedo et al., 2010; Blake, 2016; Bolhuis, 2003; Bracken, 2015; Burries, 2019; Istifci & Goksel, 2022) emphasized the importance of assigning accomplishing group interactive learning activities by children based on giving them the opportunities to discuss what they reach through their research and investigation on smart devices’ applications, which supports their learning and increases their knowledge and study achievement.

Directing children to conduct research and investigation through smart devices is associated with the role of children education female teachers in charging with performing activities relevant to science process skills through smart devices by implementing activities of observation, description and prediction during browsing electronic websites and digital applications. This confirms the importance of providing digital practical activities in digital learning environments away from frequent interruption factors of smart devices (Bradform & Hamer, 2022; Pattermann et al., 2022; Ulfayantik et al., 2022).

Digital activities based on research and investigation motivate children and encourage them to learn as a result of the diversity of means and methods of knowledge acquisition through the child’s learning independency and self-reliance, in addition to diversity and renewal in applications and easiness of searching for information and communicating with teachers, as well as asking them and discussing the research results with them directly, without postponement or delay in most cases (Al-Bagdadi, 2014; Hamdi, 2007; Khalida 2007). In this context, field studies (Darko-Adjei, 2019) revealed the role of smart devices in facilitating and enhancing the learning of students through research and investigation, where there is a strong, positive relationship between children’s learning and using smart devices. So, the findings obtained by Ameza & Baertb (2020) came to show the importance of using smart devices in a systematic, planned manner, where effective and constructive use of smart devices is directly correlated with the teacher’s role in directing the learners to the sound use of these devices.

For the sake of continuity of the strong relationship between the effective employment of smart devices and increasing children’s learning, Maguire et al. (2010) emphasized the importance of the teacher’s role which is based on guidance, counseling, behavior monitoring, fault correction and motivation system’s implementation. This role is represented in managing the children’s behavior and its sound control based on the safe use of smart devices in classrooms, as well as organizing purposeful dialogue and discussion. In this context, previous studies (Algani, 2007; Ashafei, 2005; McMahon 2015) indicated the importance of the effective role of childhood education female teachers in the sound employment of smart devices in children’s learning through logical dialogue that encourages children to think and generate numerous linguistic and mathematical concepts.

Directing children to use smart devices is not restricted to the enhancement of their knowledge and skills; it rather extends to contribute to developing emotional and social aspects, where self-confidence and spontaneous cooperation among mates increase and the accurate motor skills develop (Clark & Abbott, 2016; Papadakis et al., 2022). In a case study conducted by Rikken-Evers et al., (2022), the results revealed the existence of a big efficiency of the role of smart devices in reducing distractions in mentally disabled and improving the attention span. Also, the results showed that the effective use of smart devices enhances the level of classroom participation in mentally disabled and develops the research skills and poor fine motor skills.

In spite of the importance of smart devices and their role in achieving learning outcomes in children, previous studies (Nanni & Pusey, 2020; Ng, 2012; Omur & Cubukcu, 2017; Ozdamar-Keskin et al., 2015) showed that employing smart devices requires a control process and a plan determined by teachers and parents, where there are internal and external control factors. Internal control factors stem from the child himself/herself and his/her effort and personal features. External control is practiced by the raiser who has an influence on the individual and his/her behavior. This results in that the child recognizes the consequences of his/her conduct and that there is someone who supervises him/her and renders him/her accountable for his/her behaviors, meaning that there are certain laws and orders that should be obeyed and penalties which the child might be subjected to. This type of control is the most efficient one in controlling the behaviors of individuals and their adherence to the laws of educational institutions, among others. For smart devices to be efficiently employed, it has become necessary to find ways to control their usage inside schools for the sake of benefiting from their employment.

Also, it should be noted that it is necessary to direct children and make them aware of that over-using smartphones has negative consequences, such as sleeping disturbances, obesity and social isolation. Reckless use of the Internet can also expose children to inappropriate materials and cyberbullying through the Internet. Therefore, content filtering software should be embedded in smartphones used by children (Yadav & Chakraborty, 2021). Based on this, Yadav & Chakraborty (2021) recommended the necessity of conducting an objective assessment of the ability of children to use smartphones, as well as designing the applications according to the children’s abilities and interests in the different age groups, along with the effective use of apps in raising children accompanied by school and parental supervision of the children’s use of digital technologies.

In order to increase the effectiveness of children’s behavior while using smart devices, Asaifi (2011) indicated the necessity of establishing a constitution for all children, relevant to the sound procedures of safe employment of smart devices in the educational process, in addition to motivating and awarding children who are committed to the laws of using smart devices and follow-up the information and assignments published on education platforms; or by holding sessions and dialogues to reinforce the awareness of how to use smart devices in the best manner in the teaching-learning process; or by communicating with the children’s parents to cooperate with them and agree with them on a mechanism that enables them to employ smart devices inside and outside schools. Furthermore, it is required to make children acquainted with and aware of the importance of constructive and beneficial social interactions emanating from using smart devices away from entertainment and time wasting (Istific & Goksel, 2022; Yang & Kim 2014; Yuce, 2019; Dellori, 2006; Steffens, 2006).

Accordingly, directing children to effective employment of smart devices lies in the importance of activating children as a center of learning in the so-called children-centered learning through employing smart devices as a learning resource, which enhances the children’s learning and helps them renew, innovate and be open on the world in order to be able to form linguistic, mathematical, social, national and emotional learning experiences. So, it can be said that the current study is considered one of the rare studies in Arab environments anticipated to add new knowledge through revealing the effective use of smart devices in children’s learning in Jordan. It is expected that the findings of this study will serve as a reference for childhood education female teachers to obtain better information on the effective use of smart devices and establish applications of true learning value for children.

Based on the above, one can say that the vital role of using smart devices in children’s learning environments depends heavily on the teacher’s role in providing stimulating learning environments to enhance children’s learning, particularly because children’s use of technology does not occur in vacuum, but in the context of teaching practices. However, childhood education female teachers have contrasting visions about the effective use of smart devices in terms of presenting the educational content which is appropriate for children with focusing on the way of interaction with the content.

## Study problem and importance

The wide spread of smart devices in Jordan after the Covid-19 pandemic forms a danger to children in the absence of clear guidance from the family, the surrounding environment and the school. Children find themselves in front of voluntary or coercive excessive use of smart devices. It is noticed that there is an increase in focusing on using smart devices in recent time as a source of building and forming the children’s learning experiences.

On the other hand, there are many children who are still using smart devices in a manner which is far from the objectives of this technology. Moreover, there is a slight, nearly unnoticed employment of smart devices in the teaching-learning process in Jordan.

Based on this and because of the importance of coping with the educational advancements in light of the wide spread of smart devices in the hands of children and their intensive use of these devices, it has become necessary to employ smart devices in the teaching-learning process in a sound and safe manner based on counseling and guidance by teachers and parents, since the effective employment of smart devices is directly associated with directing children to use applications or educational platforms that encourage and motivate children and stimulate their desire to employ their smart devices to learn inside and outside the school.

In spite of the great importance of using smart devices in learning environments, the majority of previous studies in the Jordanian context have focused mainly on the effectiveness of learning of adult students through smart devices. There have been no sufficient studies that focused on the role of childhood education female teachers in directing children to the effective and sound use of smart devices, particularly in light of the fact that these devices have become available in the hands of children as an integral part of the teaching-learning process.

Since the insertion of smart devices into school learning environments is deemed to be an educational renewal that appeared with the outbreak of the Covid-19 pandemic, a gap in our knowledge had appeared about the role of childhood education female teachers in directing children towards effectively using smart devices to form and build their learning experiences, particularly since children’s learning has recently become based on the children-centered learning approach, which relies on the principle of gradual transfer from the teacher’s dominance to the child’s dominance in learning through smart devices. However, this gap is narrowed when researchers in the educational domain deal with it. The current research comes to bridge the knowledge gap in Jordan on the effective role of childhood education female teachers in directing children to employ smart devices in their learning, from the teachers’ viewpoint.

More specifically, this study attempts to answer the following question: What roles should childhood education female teachers assume in directing children to effective use of smart devices in developing their learning experiences?

## Procedures of the study

### Sample of the study

The subjects of the study were chosen from among the childhood education female teachers in north Jordan who teach at the childhood education schools (children’s age 5–9 years) in the second semester of the scholastic year 2021/2022. This sample included female teachers, not male teachers, because education in childhood was limited to female teachers only. This means that male teachers are not allowed to teach children.

The sample of the study consisted from (83) female teachers, for the purpose of conducting the semi-structured interview. This choice was carried out using the available sampling method, according to the personal desire of female teachers to participate in personal interviews. The respondents’ agreement was taken in advance and they were informed that the data presented by them will be completely confidential and exclusively used for scientific-research purposes.

### Study instrument

The current study relied on the semi-structured interview to reveal the role of childhood education female teachers in directing children to effective use of smart devices to develop their learning experiences. This instrument was prepared and developed referring to relevant literature and previous studies on the employment of smart devices in children’s learning. Accordingly, six questions were formulated which were refereed by specialists in the field of early childhood and information technology, as well as a number of teachers and supervisors in the childhood education stage. In light of the remarks of referees, two questions were eliminated and two other questions were reformulated. Accordingly, the interview consisted in its final form of four questions and this modification was carried out to ensure the validity of the study instrument. These questions are:

1. What is your opinion about the use of smart devices in children’s learning?
2. Do you think that smart devices are a major educational resource in children’s learning? How?
3. What are the best learning practices in employing smart devices in children’s learning?
4. What are your uses of smart devices in enhancing children’s learning?

The reliability of the instrument was verified through an interview that was conducted twice with six teachers from outside the research sample, where the separating span between the first and second interviews was sixteen days. Then, another analysis was conducted by specialized analysts.

To ensure the consistency among analysts, the Cooper’s equation was used to calculate the degree of agreement or divergence in the analysis. The inter-coder reliability coefficient showed a conformity of 100% agreement among the analysts. This means no discrepancies were detected in the data analysis, which indicated complete consistency between the two analyses.

In order to obtain high reliability during data collection, the qualitative research methodology was considered (Bell, 2014; Bryiman & Bell, 2018; Burton 2000; Cohen et al., 2020; Creswell, 2018; Glaser & Strauss, 2017), according to the following points:

1. The purpose and objectives were clarified to the teachers who showed a desire to participate in the interviews. Also, they were told that the information presented by them will be handled with full confidentiality and exclusively used for scientific-research purposes.
2. The participants’ agreement was obtained in advance to record the interview using audio recording.
3. Time and place of conducting the interview were determined, taking the respondent’s circumstances into account.
4. A relationship between the interviewer and the respondent was established based on respect and familiarity before starting the interview, with the aim of providing the suitable conditions to conduct the interview.
5. The interviewer avoided to know the respondents’ names, where every respondent was given a number, in order to encourage the respondents to express their ideas about the research topic.
6. The four interview questions were posed to the respondent. The accuracy level of the respondents in expressing their opinions was confirmed through asking some questions at the end of the interview in various forms that include the same ideas carried by the questions asked at the first time. This step was an indicator of the level of credibility of the study sample members’ responses.
7. The interview was discharged from the audio cassettes by the interviewer and presented to the respondent to show her opinion about what she said in the interview, giving her the opportunity to cancel or add what she deems appropriate. This step represents the best indicator of data consistency obtained by means of personal interviews.

In order to analyze the data collected by semi-structured interview, the qualitative-research analysis methodology was used, represented in the grounded theory approach, where the ideas which appeared from the study data through the interviews were adopted and the features or categories were reached through:

- Downloading the interviews onto paper separately.
- Examining reading of every word, phrase or sentence mentioned by the subjects of the study.
- Adopting the encoding of responses.
- Putting similar or nearly similar ideas in sub-categories under main categories.
- Verifying the consistency of data analysis through letting two persons carry out the analysis process. This process revealed full consistency between the two analysts according to the main categories and sub-categories, which confirms the accuracy of the analysis process.
- Calculating frequencies and percentages of responses as distributed within the sub-categories.

## Findings of the study

This study aimed at revealing the role of childhood education female teachers in directing children to effective use of smart devices to enhance their learning experiences. To achieve this aim, the responses of the study sample members in the semi-structured interview were analyzed. The analysis results showed that there is a big role of childhood education female teachers in directing children to effective use of smart devices. The responses can be presented in six main categories, as follows:

- Directing children to organize their self-learning during using smart devices:
- Directing children to acquire digital social interaction skills.
- Directing children to learn innovation during using smart devices.
- Directing children to avoid the harms of smart devices through preventive guidance.
- Directing children to participate in digital activities.
- Directing children to learn through purposeful digital applications.

These categories can be demonstrated as follows:

**First: Directing children to organize their self-learning during using smart devices**.

The interview data analysis results found out six sub-categories related to the role of childhood education female teachers in directing children to organize their self-learning during using smart devices, as depicted in Table 1.

**Table 1:** Frequencies and percentages of the role of childhood education female teachers in directing children to organize their self-learning during using smart devices

No.	Sub-categories	Frequency (N)	Percentage (%)
1	Directing children to self-answer questions through digital research and investigation	80	96.38
2	Providing children with electronic applications to perform self-learning tasks	77	92.77
3	Encouraging children to bear self-responsibility through implementing digital activities	73	87.95
4	Presenting guidance leaflets for parents to help their children organize their digital self-learning	70	84.43
5	Reinforcing the children-centered learning approach through learning tasks based on reading electronic stories	69	83.13
6	Presenting encouragement motivations to children who use smart deices for educational purposes	67	80.72

The results shown in Table 1 indicate that the role of childhood education female teachers contributes to enabling children to practice self-organization of their learning, where it is focused on digital applications for self-learning. The responses of the respondents showed that 96.38% of them believe that building children abilities of self-organization is considered a main aspect of enabling children to become self-organized through research and investigation using smart devices. In this context, some responses of the respondents can be quoted as follows:

*“Yes, the role of childhood teacher is big and important in enabling children to self-organize themselves. I [the respondent] focus on continuous communication with children to use smart devices in their learning, especially in guiding them to research and investigation…*.

*I charge children to perform self-learning tasks to be electronically implemented…*.

*I endeavor to enable children to practice self-learning…. It is important to focus on the child to benefit from smart devices…. We focus in our school on disseminating the learning culture in the childhood through modern technologies…. We direct children to learn self-organization skills through smart devices*.

*We concentrate on training the child to bear the responsibility of his/her learning to be independent in his/her learning through smart devices”.*

The responses above reveal that the subjects of the study are clearly aware of numerous roles on how to utilize smart devices to develop self-organization in children through various digital practices. This appears obviously through their expressions that indicate the effective teacher’s role in directing children correctly to learn through smart devices.

**Second: Directing children to acquire digital social interaction skills.**

The analysis of the interview data revealed the existence of eight sub-categories pertinent to the role of childhood education female teachers in directing children to acquire digital social interaction skills. Table 2 shows the results.

**Table 1:** Table 2. Frequencies and percentages of the role of female teachers in directing children to acquire digital interaction skills

No.	Sub-categories	Frequency (N)	Percentage (%)
1	Reinforcing different types of digital communication between children and educational and administrative staff in the school	78	94.00
2	Encouraging children to participate in local and international digital learning platforms	70	84.43
3	Providing children with instantaneous feedback through smart devices	67	80.72
4	Encouraging digital conversations between the teacher and the children	67	80.72
5	Using the flipped-learning strategy that allows children to interact socially with their classmates	60	72.29
6	Providing children with educational programs to reinforce social interaction among children themselves through smart devices	53	63.86
7	Encouraging children to perform exploratory trips through the web quest strategy	50	60.24
8	Allowing children to participate in taking decisions through smart devices	47	56.63

There was a diversity in the responses of the study sample members in ascertaining their roles in reinforcing the children’s abilities in digital interaction through smart devices, by means of constructive interaction between children and the others in the local and international school society. Some respondents have expressed this as follows:

“*We in our school do our best to let children refrain from social isolation that could accompany the use of smart devices, through integrating children in social interactions by electronic communication.*

*I (the respondent) direct children to acquire digital social interaction skills through digital activities that are capable of enabling the child to express his/her ideas and feelings…. All this is positively reflected on the development of interactive experiences in the childhood.*

*The role of childhood education teacher is very important in activating smart devices as a source of learning and a learning method through flipped learning and web quest strategies…. After employing these strategies, interactions with children are held.*

*I (the respondent) communicate with my children through digital devices to provide them with feedback about their learning… and encourage them to take decisions pertinent to ideas they reach through group discussions*”.

The responses above reveal that the participants in the study are clearly aware of their instructional roles in activating smart devices as a teaching-learning source, with the aim of reinforcing the aspects of digital social interaction in children to allow them to get rid of social isolation that could accompany the use of smart devices. These results give indicators with considerable value of the teacher’s role in enhancing the children’s learning through smart devices.

**Third: Directing children to learn innovation during using smart devices**.

The interview data analysis results revealed six sub-categories related to the role of childhood education female teachers in directing children to learn innovation during using smart devices, as illustrated in Table 3.

**Table 3:** Frequencies and percentages of the role of female teachers in directing children to learn innovation during using smart devices

No.	Sub-categories	Frequency (N)	Percentage (%)
1	Giving children innovative-thinking activities based on using smart devices	80	96.38
2	Encouraging children to discover and produce knowledge through employing digital knowledge vessels	71	85.54
3	Reinforcing children’s innovations through practicing digital games	66	79.51
4	Disseminating innovation culture among children through using smart devices	59	71.08
5	Introducing digital innovative educational programs to children	50	60.24
6	Inviting specialists to present lectures on innovation using smart devices	45	54.21

Table 3 above shows that the analysis of interview results confirms that using smart devices is considered as an important approach in directing children to be innovators in their learning. The analysis results shows that childhood education female teachers endeavor to disseminate the culture of using smart devices among children as a source of knowledge discovery and building through practicing various digital activities. This is confirmed by the fact that 96.38% of the respondents mentioned the importance of providing children with innovative-thinking activities based on using smart devices, as well as reinforcing these innovations to be a practiced behavior in the children’s daily life. In this context, some of the sample members’ expressions can be quoted as follows:

*I [the respondent] focus on how to design digital activities that encourage children to practice creative thinking.*

*I disagree with the opinions of some people who say that smart devices do not encourage innovation…. I encourage children to practice creative thinking through selecting digital games that push the child to practice innovative thinking.*

*I discovered the children’s talents and hobbies using smart devices through their expressions in national festivals.*

*Most of my teaching is based on directing children to interact with digital applications that push the child to produce genuine and new knowledge through answering questions that measure the child’s higher skills…. I ask the child to criticize characters through reading digital stories…. I ask children to express their opinions about some characters on the children-learning sites*.

From Table 3, one can conclude the various justifications that present practical evidences on the aspects of effective employment of smart devices in developing the children’s learning experiences, since they are considered as a starting point to developing innovative thinking. It is clearly obvious from the responses that employing smart devices is one of the most prominent concurrent orientations in reinforcing innovations among children, so that their use becomes an approach to the reinforcement of innovation as well as an innovation-incubating environment, making the effective use of smart devices one of the forms of children’s culture.

**Fourth: Directing children to avoid the harms of smart devices through preventive guidance**.

The results of the interview data revealed six sub-categories related to the role of childhood education female teachers in directing children to avoid the harms of smart devices through preventive guidance. Table 4 shows these sub-categories arranged in descending order according to their frequencies.

**Table 4:** Frequencies and percentages of the role of female teachers in directing children to avoid the harms of smart devices through preventive guidance

No.	Sub-categories	Frequency (N)	Percentage (%)
1	Getting children aware of the health harms resulting from addiction to using smart devices	82	98.79
2	Holding individual dialogues between the administrative and teaching staff and the children about the safe use of smart devices	80	96.38
3	Inviting the educational psychological counselor in the school to disseminate the culture of safe use of smart devices	64	77.10
4	Presenting awareness leaflets to parents to make their children aware of the safe use of smart devices	61	73.49
5	Presenting daily awareness advices through school broadcasting on the safe use of smart devices	59	71.08
6	Using clarifying means such as cartoon drawings that point out the right and wrong behaviors in using smart devices	49	59.03

The results reveal that one of the aspects of progress in directing children to use smart devices is getting them aware of harms resulting from addiction to using smart devices, in addition to showing them how they can use smart devices in a safe manner through preventive guidance and awareness leaflets for parents, as well as pointing out the role of school broadcasting presented to children every morning. The following quotations express the study sample members’ responses.

“*I (the respondent) encourage children to use smart devices for the purpose of learning linguistic and mathematical skills, but at the same time, I train them on the safe use of smart devices…. Some of my children have talked to their parents who adviced them about the correct use and the correct sitting position when using smart devices.*

*I really discuss with my children in the class and we talk about the safe use of smart devices through real stories that suit their mental level.*

*In our school, we have a protocol for distributing weekly awareness leaflets on the safe use of smart devices.*

*We put posters, pictures and cartoon drawings on the school walls to show the safe use of smart devices.*

*We charge children to criticize incorrect uses of smart devices through class discussions*”.

**Fifth: Directing children to participate in digital activities**.

The interview data analysis results reveal four sub-categories related to the role of childhood education female teachers in directing children and encouraging them to participate in digital activities, as depicted in Table 5.

**Table 5:** Frequencies and percentages of the role of female teachers in directing children to participate in digital activities

No.	Sub-categories	Frequency (N)	Percentage (%)
1	Encouraging children to participate in all digital school events held by the school administration	81	97.59
2	Integrating curricular digital activities within school activities so that children participate in them due to their hobbies	75	90.36
3	Encouraging children to participate in local and international virtual childhood meetings and conferences	45	54.21
4	Endorsing a school semester award granted to children participating in digital learning activities	40	48.19

The results show that childhood education female teachers have huge contributions to the employment of smart devices as a main source of practicing school activities, so that children are integrated in different forms of activities according to their hobbies and desires. This was expressed in the interviews with the study sample members. Some citations of the responses can be shown as follows.

“*I encourage children to participate in international meetings, such as the International Childhood Meeting and the Children Meeting in Abu Dhabi through virtual participations.*

*I encourage children to participate in digital school activities that support and reinforce English language curricula.*

*I directed my children to present electronic activities in childhood meetings…. My children have won in drawing and building activities through using smart devices.*

*I encourage my children to participate in the school reward on the best production of drawings and pictures that support positive environmental behaviors.*

*I encourage my children to participate in presenting digital stories during using smart devices…. My aim of doing so is to reinforce self-confidence among my children*”.

From the responses quoted above, it is clear that childhood education female teachers practice critical roles in reinforcing the children’s usage of smart devices through encouraging them to participate in different local and international digital events, with the aim of developing the child’s character to be a partner in and a producer of knowledge rather than being a consumer of it.

**Sixth: Directing children to learn through purposeful digital applications**.

The interview data analysis results revealed five sub-categories related to the role of childhood education female teachers in directing children to learn through purposeful digital applications, as depicted in Table 6.

**Table 6:** Frequencies and percentages of the role of female teachers in directing children to learn through purposeful digital applications

No.	Sub-categories	Frequency (N)	Percentage (%)
1	Selecting applications of the ability to develop linguistic skills, such as touch screen app. supported with language	77	92.77
2	Downloading applications that are suitable to the curriculum’s content with the aim of achieving learning outcomes	70	84.33
3	Focusing on drawing and building activities	67	80.72
4	Directing children to learn through interactive games, particularly those pertinent to concepts of mathematics and science	65	78.31
5	Selecting attractive and enjoyable electronic stories that suit the children’s growth characteristics	55	66.26

The responses show that childhood education female teachers have big contributions to the employment of smart devices as a main source of developing the basic learning skills in language, mathematics and science, noting that these skills form the essence and core of childhood curriculum in the Jordanian environment, where directing children to correct use of smart devices effectively contributes to developing the children’s learning experiences.

Some respondents expressed that as shown in the following citations.

*It is correct that touching screens can generate joy…. I really direct my children to applications that lead to the growth of mental skills through interaction with the language of digital games.*

*My role as a children’s teacher is helping them download applications to train children on resolving mathematical puzzles…. My children do not use games for entertainment, but to achieve certain learning outcomes.*

*I directed my children to practice different digital games to get trained on acquiring language skills, where I hold discussions with my children after they have practiced the games*.

The quotations above demonstrate that childhood education female teachers practice very valuable roles that focus on directing children to use various applications that achieve learning outcomes in language, mathematics and science with focus on achieving the joy of learning through electronic stories that suit the children’s nature and capabilities.

## Discussion of the findings

The findings of the study revealed that childhood education female teachers have roles of great value in directing children to effective use of smart devices to develop their learning experiences. It was clear that childhood education female teachers direct children to use smart devices for the development of their self-organization. This result confirms that employing smart devices in developing the children’s experiences aims at making the child an active partner in the process of his/her learning, so that his/her ability to discuss and conduct dialogue grows. This result is consistent with the educational international trends that point out the role of information and communication technology in developing self-skills among children, which is reflected on the academic, social and emotional efficacy. In this context, previous studies (Burris, 2019; Istifci & Goksel, 2022; Yang & Kim, 2014; Yuce, 2019) have indicated that employing learning through smart devices has a great role in developing self-organization and self-confidence skills. It also contributes to the development of different forms of learning experiences among children, since it enables children to express their ideas and feelings and understand the ideas and feelings of others.

These results present an empirical evidence on that children will become skillful learners in reaching knowledge through different forms of digital communication, in addition to enabling them to assess their knowledge, employ it and exchange it in various life contexts. This was confirmed by the response of one of the subjects of the study who said:

*We in our school want to prepare children for the future to be able to reach, build and employ knowledge using digital communication means away from receiving knowledge through memorization*.

This result is consistent with those of previous studies (Darko-Adjei, 2019; Liu et al., 2021; Nikolayev et al., 2022) that pointed out that the use of smartphones plays a great role in influencing the children’s learning experiences through various digital educational activities.

Moreover, the results revealed that childhood education female teachers practice preventive guidance roles in directing children toward the safe use of smart devices. These roles are represented in clarifying the health and psychological harms resulting from addiction to the use of smart devices, through conducting dialogues and discussions with children about the safe use of smart devices. In order to disseminate the culture of safe use of smart devices, awareness leaflets and advices as well as cartoon drawings are presented that express the safe use of smart devices.

This role of childhood education female teachers is deemed to be a central role in directing, advising and guiding children using different means and preventive measures that contribute to protecting childhood. This result is consistent with the concurrent educational trends (Cristia & Seidl, 2015; Nikolayev et al., 2022; Paudel et al., 2017; Shamsah et al., 2022; Radesky et al., 2015; Yadav & Chakraborty, 2021) that emphasize the role of school education in directing children to the safe use of smart devices, particularly since the first years of the child’s age are considered a critical stage in building and developing his/her character in different cognitive, social, emotional and health aspects.

Also, the results showed that the great role of childhood education female teachers is centered around the selection of digital applications which are based on activities that stimulate the child’s motivation to learn in an exciting manner instead of learning by receiving knowledge for memorization which is inappropriate in the normal condition. Therefore, it is important to concentrate on enabling the child to build safe relationships with smart devices and establish healthy behaviors. This result reveals the importance of the selection of high-quality applications by childhood education female teachers, as well as the monitoring of the children’s media content, since the inappropriate use of smart devices could negatively affect the children and restrict their constructive interaction with smart devices, in addition to scattering their attention. This result confirms the big importance of selecting developmentally appropriate applications and high-quality educational applications.

The study results showed that integrating digital activities as an integral part of the scientific, mathematical and social school activities forms a main factor in investing learning through smart devices by enabling the child to acquire the basic skills, such as problem solving and exploring the children’s natural environment, in addition to their interaction with friends and care givers and playing in systematic innovative ways. This direction to use smart devices is consistent with the general bases and principles of the child’s growth and development, his/her characteristics and capabilities and his/her mechanisms of learning. These effective roles of childhood education female teachers contribute to enabling children to learn through practical use of smart devices; i.e., through direct self-interaction with various applications to self-acquire knowledge through research and investigation processes, as well as problem solving and other strategies that employ the child’s higher mental skills. This leads to meaningful learning which is transferable from a context to another.

This finding confirms that the child’s role in learning is the core and center of the teaching-learning process, while the teacher’s role lies in facilitating the learning process, meaning that focus is placed on learning rather than teaching. This means that the teacher’s role revolves around planning and organizing the learning experiences, where the child achieves acquiring knowledge through his/her interaction according to the objectives of every experience.

Furthermore, the study results revealed that directing children to use smart devices focused on the children’s learning of linguistic, mathematical and science concepts and learning the arts of drawing and building. This depends on the effective design of smart devices’ applications that suit the children. It is clear from the results that the teacher plays an essential role in supporting children with educational means through applications that encourage children to learn in an atmosphere of joy, particularly since children enjoy learning through digital devices.

This result can be interpreted by the presence of awareness among childhood education female teachers of the correct direction of children to achieve learning outcomes through interactive games’ applications, drawing and building tools and benefiting from colored electronic books, electronic animations and interactive features. So, downloading various applications by childhood education female teachers through which children learn language, mathematics and science is consistent with the findings of previous studies (Jusoh, 2017; Liu et al., 2021; Radesky et al., 2015) that showed the great role pertinent to the dealing of children with smart devices’ applications in reinforcing attention among children during their learning with interactive games and drawing tools’ applications.

The study results also revealed that correct direction of children to use smart devices depends on the teacher’s ability to enable children to learn skills and strategies, as well as training them while they are in the school on the mechanisms of accomplishing clearly assigned tasks, considering diversity, with the cooperation with others or individually, which should occur on days when children do not come to the school or kindergarten, noting that the greater portion of the educational process depends on remote learning, not on electronic learning. Then, these tasks become a basic alternative.

In addition, the study results showed that the correct direction of children to use smart devices depends on communication between children and teachers through electronic applications, within small groups, individually or with parents, where interactive activities are implemented, such as interactive group dialogues, narrating and discussing digital stories, sending enriching recordings (audio and video recordings) and applied work papers from time to time, noting that the child learns only through active interaction. This study’s results can be attributed to the presence of clear views among childhood education female teachers about the importance and role of smart devices in investing the children’s abilities through active interactive learning, self-learning and learning through exploration, research and investigation away from receiving knowledge for memorization.

In light of the current study’s results, it can be said that childhood education female teachers have a central role in planning learning through smart devices. This role is represented in enabling children to execute numerous effective and constructive social interactions through activities, tasks and projects that cover the educational content of childhood curriculum.

## Conclusions, limitations, implications and recommendations

The current study aimed to uncover the role of childhood education female teachers in directing children to the effective use of smart devices in developing their learning experiences. The study used the semi-structured interview to collect data. The collected data was analyzed using the grounded theory approach.

The study results were restricted by a set of limitations that open horizons for future research. One of these limitations is that the data collection was restricted to an available sample of childhood education female teachers in north Jordan, which hinders the generalization of the results over a wider scope. So, future research should consider collecting data from south and middle Jordan, which allows better generalization of the results. Further, data collection relied on the semi-structured interview. To go deeper into the role of childhood education female teachers in directing children to effective use of smart devices, future research should rely on class observation with the purpose of observing models of children’s use of smart devices.

In light of the study results, it can be concluded that childhood education female teachers consider touching the screens of smart devices by children as a main source of learning, considerably contributing to the transmission of education from face-to-face education to electronic education. Consequently, the study results ascertain that directing children to learn with smart devices has become one of the dominant educational approaches in childhood education.

Moreover, the study concluded that childhood education female teachers attempt through directing children to use the applications of smart devices to focus on applications that suit the child’s age for the development of his/her learning experiences in the knowledge, emotional, social and skill aspects, among others. This conclusion shows that using smart devices does not depend on making and receiving calls. Rather, effective use of smart devices focuses on reinforcing the children’s abilities to be capable of browsing, sending and receiving e-mail messages, watching educational videos, examining pictures, exchanging pictures and video clips, participating in local and international learning platforms and executing various digital activities. This conclusion also shows that directing children to correct use of smart devices plays a significant role in enabling children to develop their self-organization skills as well as their interaction and innovation skills, in addition to developing the children’s self-efficacy through practicing planned, systematic digital activities in a purposeful and meaning manner rather than marginally or arbitrarily.

In light of the collective results of this study, it can be concluded that although children practice the skills of self-organization, innovation, self-interaction and social interaction, they still need to be directed by their teachers and parents to help them continue their self-learning outside the school learning environments, also known as out-door learning. So, it can be recommended that it is important to enable children to benefit from digital learning platforms, so that they are able to access them, noting that some of these platforms are not available to all children in Jordan. Moreover, it can be argued that effective use of smart devices remarkably contributes to the acquisition of children of a digital culture that could increase their awareness of various learning experiences, through their innovations that are based on producing and generating knowledge through strategies that stimulate scientific thinking, in addition to their self-interactions, social interactions and employing self-organized learning strategies by means of practicing digital activities that lead children to participate in Internet symposia, electronic symposia and digital platforms. Accordingly, the current study calls for conducting research on the efficiency of digital culture in developing the children’s experiences in language and mathematics, as well as investigating the relationship between practicing self-learning skills and digital culture.

The findings of the current study allow to conclude that the roles of childhood education female teachers in directing children to correct use of the applications of smart devices are consistent with the international trends to employ digital technology as a main source of learning with the aim of achieving various learning outcomes pertinent to the different knowledge, emotional, social and skill aspects of the child’s character, among others. The study results also reflect that childhood education teachers have directed children to use the screens of smart devices in developing their language skills through interaction with others and understanding them.

Furthermore, the study revealed that the great role of childhood education female teachers is represented in selecting the applications that suit the children’s growth characteristics for enabling the children to build and form the concepts of mathematics and science. This conclusion is consistent with the international trends in enabling the children to develop their skills in science, technology, engineering and mathematics (STEM), where this aspect is very important in developing the children’s characters to be partners in science, technology, engineering and mathematics with the aim to be prepared to face the challenges associated with these domains in the future. In short, the following instructional implications and recommendations can be presented.

1. The child’s learning through smart devices should occur in a planned manner; through interaction and movement rather than sitting and receiving information from the teacher. When this condition is fulfilled, involvement happens, learning becomes enjoying and smart devices become a driver of motivating children to practice active learning.
2. Directing children to the effective use of smart devices should not be through applications of digital technology that are based on abstract knowledge and abstract mental processes. Rather, children should be subjected to applications of smart devices that are based on learning with tangibles, like drawing and building applications, interactive digital games, as well as learning through voice, picture, movement, … etc.
3. Directing children to use smart devices should rely on applications that are suitable to the children’s growth abilities, focusing on selecting higher-quality applications and those that consider the ability of the child to concentrate and pay attention, noting that this ability is short-termed with slight differences in its lasting period among children due to age. It is worth mentioning that the period of concentration and attention in the children’s use of smart devices increases when the child is interested in the learning content, enjoys it and is active in learning from it.
4. Childhood education schools should hold meetings with parents and care givers, within small groups, in order to clarify their central role in directing children to use smart devices according to their growth characteristics, planning together to build a mechanism of continuous communication, as well as providing parents and care givers with awareness leaflets to direct their children to safe and effective use of smart devices.
5. Guidance leaflets should be issued to be used by parents and care givers to direct their children to avoid health and psychological harms caused by wrong use of smart devices, noting that these harms could affect mental, social, emotional and motor-sensory growth of children.
6. The correct direction of children to learn through smart devices should be based on the approach of active interactive learning, self-learning and learning through scientific research and investigation, noting that in this approach, the child is self-pushed to learn with strength and desire, enjoying what he/she learns, as this is the only way to benefit from digital technology in developing the children’s learning experiences.

## Data Availability

the raw data supporting the conclusions of this article will be made available by the authors, without undue reservation.
